# P58 Access to clear information on infection and antimicrobial resistance: an unmet need for cancer patients at risk of, or experiencing, infection

**DOI:** 10.1093/jacamr/dlaf118.065

**Published:** 2025-07-14

**Authors:** Josephine Owen, Denise Hunt, Gabrielle Higgins, Allison Neugebauer, Gillian Kiely, Zeshan Riaz

**Affiliations:** Pfizer Ltd, Tadworth, UK; Pfizer Ltd, Tadworth, UK; Pfizer Ltd, Tadworth, UK; Pfizer Ltd, Tadworth, UK; Pfizer Ltd, Tadworth, UK; Pfizer Ltd, Tadworth, UK

## Abstract

**Background:**

Antimicrobial resistance (AMR) poses a serious threat to human health. AMR burden is increasing globally, with 39.1 million deaths forecast from 2025–2050.^1^ Infection is amongst the most common complications experienced by cancer patients, with MDR infections occurring more frequently in cancer patients than any other group.^2^ There is a clear unmet need to better understand the perspectives of people affected by cancer, in particular around infection risk and AMR.

**Objectives:**

To gather insights of cancer patients and their carers regarding the physical and psychological burden experienced due to elevated risk of infection and AMR throughout their cancer journey, with a focus on their perspectives in relation to unmet needs.

**Methods:**

The Cancer Insights Panel (CIP) was an initiative consisting of several panel meetings that ran over 18 months to gather the perspectives of people impacted by cancer, including patients and their family and caregivers. Participant recruitment for CIP involved online questionnaires (disseminated via social media channels) designed to capture demographic information to ensure panel diversity. An ad-hoc panel, with 16 participants, was convened on 17 October 2024, to understand participant perspectives around infection risk and AMR. Participants for this panel were selected from the existing CIP cohort. Mind maps were sent out ahead of the meeting to garner initial insights. Subsequently, panel members were sent a pre-read based on the mind map feedback, to prepare them for the meeting and allow for a productive discussion into infection risk and AMR. Insights from the meeting were recorded in the Patient Experience data catalogue and thematically analysed.

**Results:**

Access to trustworthy and accurate Information around infection was identified as an overarching need for cancer patients. Key areas of interest included risk of infection during cancer treatment, how to avoid infection, and what symptoms to look out for should infection manifest. Additionally, the insights highlighted a need for better information and support for cancer patients when communicating about infection risk to their peers and support networks. The term ‘antimicrobial resistance’ was poorly understood amongst participants often resulting in confusion and fear surrounding antimicrobial use and AMR risk. Furthermore, elevated risk of infection and AMR were associated with mental health burden, with the panel noting that mental health information and support were not always accessible. Inconsistencies in co-ordination of healthcare and communication between healthcare providers also contributed to mental health burden, ‘the information I was given in appointments with different consultants seemed conflicting… this indecision about treatment was a very anxious time for me… I was so frightened and upset’.

**Conclusions:**

The CIP demonstrated that participants feel improving information around infection and AMR is crucial, to help mitigate anxiety and stress. Better quality, delivery and access to trustworthy information would help address this unmet need and enhance the patient journey.

## References

[1] GBD 2021 Antimicrobial Resistance Collaborators . Global burden of bacterial antimicrobial resistance 1990–2021: a systematic analysis with forecasts to 2050. Lancet 2024; 404: 1199–226. 39299261 10.1016/S0140-6736(24)01867-1 PMC11718157

[2] ReAct Group , Successful Cancer Treatment Relies on Effective Antibiotics. 2020. https://www.reactgroup.org/wp-content/uploads/2020/05/Successful_cancer_treatment_relies_on_effective_antibiotics_ReAct_Policy_Brief_May_2020_web.pdf

